# Efficacy of Robot-Assisted Gait Training Combined with Robotic Balance Training in Subacute Stroke Patients: A Randomized Clinical Trial

**DOI:** 10.3390/jcm11175162

**Published:** 2022-08-31

**Authors:** Irene Aprile, Carmela Conte, Arianna Cruciani, Cristiano Pecchioli, Letizia Castelli, Sabina Insalaco, Marco Germanotta, Chiara Iacovelli

**Affiliations:** 1 IRCCS Fondazione Don Carlo Gnocchi, 50143 Florence, Italy; 2 Laboratorio di Analisi del Movimento, Policlinico Italia Piazza del Campidano 6, 00162 Rome, Italy; 3 High Intensity Neurorehabilitation Unit, Fondazione Policlinico Universitario A. Gemelli IRCCS, Largo Francesco Vito 1, 00168 Rome, Italy; 4 Department of Neuroscience, IRCCS San Raffaele Pisana, 00166 Rome, Italy; 5 Department of Aging, Neurological, Orthopaedic and Head-Neck Sciences, Fondazione Policlinico Universitario A. Gemelli IRCCS, Largo Francesco Vito 1, 00168 Rome, Italy; 6 Rehabilitation and Physical Medicine Unit, Fondazione Policlinico Universitario A. Gemelli IRCCS, Largo Francesco Vito 1, 00168 Rome, Italy

**Keywords:** stroke, robot-assisted gait training, balance, end-effector device, rehabilitation

## Abstract

Recently, the use of robotic technology in gait and balance rehabilitation of stroke patients has been introduced, with positive results. The purpose of this study was to evaluate the effectiveness of robotic gait and trunk rehabilitation compared to robotic gait training alone on balance, activities, and participation measures in patients with subacute stroke. The study was a randomized, controlled, single blind, parallel group clinical trial. Thirty-six patients with first ischemic or hemorrhagic stroke event were enrolled, and they were randomized in two groups: Gait Group (GG), where they received only robotic treatment for gait rehabilitation through an end-effector system, and Gait/Trunk Group (GTG) where they performed end-effector gait rehabilitation and balance with a robotic platform, 3 times/week for 12 sessions/month. At the end of the study, there was an improvement in balance ability in both groups. Instead, the lower limb muscle strength and muscle tone significantly improved only in the GTG group, where we found a significant reduction in the trunk oscillations and displacement during dynamic exercises more than the GG group. The robotic platform which was added to the gait robotic treatment offers more intense and controlled training of the trunk that positively influences the tone and strength of lower limb muscles.

## 1. Introduction

Stroke is the largest cause of adult disability worldwide, with a significant impact on individuals, their families, and cost for society [1]. Post-stroke disability results in a health care problem and has many long-term consequences involving human mobility and balance [2]. In general, patients learn to walk independently within six months after a stroke event. Therefore, recovery of ambulation can be considered a realistic goal of the rehabilitation program of almost all patients after a stroke [3,4]. Furthermore, many studies indicate that walking and balance problems persist in the chronic phase after the initial illness, showing that walking dysfunction can have a significant impact on patients’ quality of life [3,5,6]. Consequently, restoring and improving walking function is a primary concern to obtain independence in daily life [7], and improving the daily quality of life [8].

In recent years, efforts are being made to make rehabilitation treatment more useful for patients; several works highlight the effectiveness of combined robotic and non-robotic rehabilitation treatment to improve motor and cognitive function in patients with stroke [9,10]. The introduction of robotic technology in walking rehabilitation of stroke patients has attracted great interest due to intensive and repetitive task practice enriched with multi-sensory stimuli [11]. Moreover, robotics rehabilitation makes it possible to objectively and quantitatively assess the patient’s disability and disease progression and reduce the physical burden on the therapist [12].

Robot-Assisted Gait Training (RAGT) can be performed with both end-effector and exoskeleton devices [13]. Either exoskeleton or end-effector robots have been used for gait training in neurological disorders [14,15,16,17,18,19,20]. Some studies have been conducted to evaluate the effects of RAGT compared to conventional walking rehabilitation in stroke patients [2,21,22,23]. However, neither the superiority of RAGT training over conventional training nor the fact that RAGT training promotes a safer gait when compared to conventional training has been demonstrated. Indeed, one consequence of suboptimal gait recovery in stroke patients is the high risk of falls, which worsens their quality of life [24,25,26]. Recovery of a more fluid, safe, and correct gait is a fundamental prerequisite for patients’ independence in activities of daily living, as well as recovery of dynamic balance. Therefore, balance and walking should be treated trough an appropriate rehabilitation program.

Although various interventions have been proposed to improve balance following the stroke, they are heterogeneous in some settings, duration, intensity, and type of interventions or device used, and no one intervention has been established as superior to others [27,28]. A recent review showed that there are still unexplored paths in the robot-assisted lower-limb in stroke, suggesting combination approaches that may be more efficacious than a single approach [29]. To date, only “case studies” seem to investigate the effects of a combined waling and balance training in stroke patients [30,31], using a gait assisted device. In these studies, the post-stroke patients after sessions of balance-perturbed training using a treadmill for walking training improved gait symmetry, push-off, and timing as well as the response strategy to perturbations.

Recently, robots have been developed for balance rehabilitation, opening up the possibility of using robotics for balance recovery [32,33]. The robotic approach could be useful for dynamic balance recovery, which is very important during gait and crucial for achieving safer walking.

The hypothesis of this study is that combined robotic walking and balance training improves balance, walking ability, and quality of life more than the robotic gait treatment alone. Therefore, the study aimed to evaluate the effectiveness of robotic walking *plus* trunk rehabilitation compared with robotic walking training alone on balance, activity, and participation measures in subacute stroke patients.

## 2. Materials and Methods

### 2.1. Study Design and Participants’ Recruitment

This is a randomized (1:1 ratio), controlled, parallel-group clinical trial in subacute stroke patients comparing end-effector robotic gait training combined with robotic balance training vs. end-effector robotic gait training alone. The study was conducted in the Fondazione Don Carlo Gnocchi of Rome and it was registered with ClinicalTrials.gov (NCT04162197).

Inclusion criteria: subjects with first cerebral stroke; 2 weeks up to 6 months post the acute event (subacute patients); age between 18–85 years; able to adapt to end-effector footplates without significant limitation of joint range of motion, able to give written consent, and comply with the study procedures.

Exclusion Criteria: contractures of the hip, knee, or ankle joints that limit range of motion during ambulation; medical issues that preclude full ambulation and weight bearing (e.g., orthopedic injuries, pain, severe osteoporosis, or severe spasticity); cognitive and/or communicative disability (e.g., due to brain injury); inability to understand required study instructions; heart pathologies; and anxiety or psychosis that could interfere with the use of the equipment or test.

Written informed consent was obtained from each subject. Ethical approval of the treatment and an evaluation protocol was granted by the institutional Ethics and Experimental Research Committee of the Fondazione Don Carlo Gnocchi (date: 13 March 2019; FDG_13.3.2019).

### 2.2. Randomization

**Figure 1:**
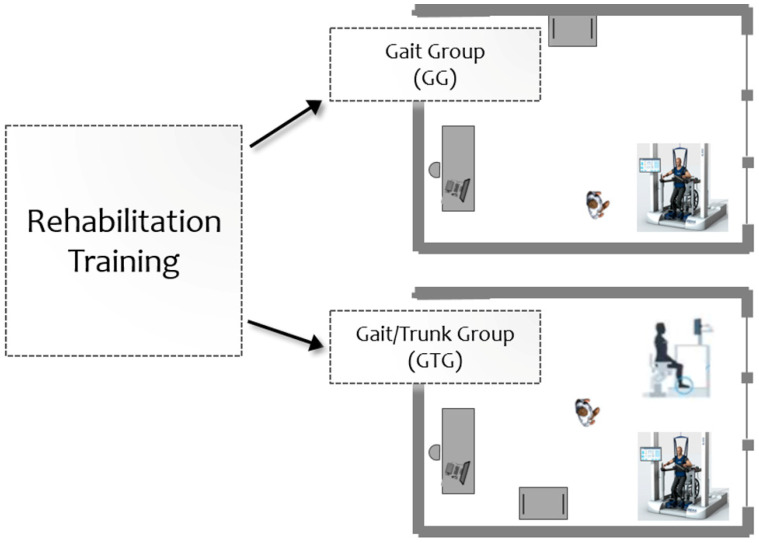
Rehabilitation training: Gait Group (GG) and Gait/Trunk Group (GTG).

Patients were randomized into two groups and conducted two different types of rehabilitation training: one group performed gait training using a robotic end-effector device for RAGT (Gait Group, GG); the other group received a combined robotic treatment program with the robotic end-effector system for gait and a robotic platform (Gait/Trunk Group, GTG) (Figure 1).

Randomization sequence was generated by using the R (version 3.3.0, R Core Team, Vienna, Austria) package blockrand, with random block sizes ranging from 2 to 8. Randomization was stratified according to the Ambulation Index value (0–3 severe disability; 4–7 low disability) so that the subjects in each group were closely matched; a researcher with no clinical role in the study prepared the randomization list. An independent observer, blinded to the intervention performed in the protocol, evaluated all participants.

### 2.3. Therapeutic Interventions

GG includes patients who received RAGT treatment only, 3 times/week for 12 sessions/month. The RAGT is a robotic gait device (G-EO system; Reha TechnologyAG; Olten, Switzerland), and end-effector characterized by a Body Weight Support (BWS) and 2 footplates placed on a double crank and a rocker gear system, with 3 Degrees of Freedom (DoF) each, which allow the step length and height to be controlled. The trajectories of the footplates and the vertical and horizontal movements of the center of mass were fully programmable, thus allowing the simulated walking on the ground to be simulated repetitively. During training, patients were asked to walk, at a varying speed, for 45 min and with partial BWS. Participants started with 30–40% BWS and an initial speed of 1.5 km/h; they increased to a maximum of between 2.2–2.5 km/ h and reduced the initial BWS to 15%. The therapist remained in front of the patient during the treatment session to provide any help if needed. Over 45 min, the patient simulated a minimum of 300 steps [34]; patients were allowed to rest during the session, although they were asked to walk continuously for a minimum of 5 min during each session.

GTG patients received a combined robotic treatment program with G-EO system and a robotic platform (Hunova, Movendo Technology srl, Genoa, IT), 3 times/week for 12 sessions/month. The robotic platform has two 2-DOF actuated and sensorized platforms located under the seat (“seat platform”) and at floor level (“floor platform”) that induce multidirectional movements to improve postural stability. In general, the two movable platforms allow physical activity to be performed in both seated and standing positions, in mono- and bi-podal conditions. The robotic device is paired with a wireless inertial sensor (Inertial Movement Unit—IMU) placed on the patient’s trunk, at the level of the sternum, which allows it to record the subject’s trunk movements. The inertial sensor includes an accelerometer, a gyroscope, and a magnetometer. In this way, it measures the rotations along the *x*-axes (Roll) and *y*-axes (Pitch) and evaluates the patient’s trunk movements in the frontal and sagittal planes, as well as movement accelerations.

The device can operate in passive, active, and assistive mode, detecting compensatory trunk movements giving sensory feedback during exercise.

Graphic applications match the proposed exercises, promoting the patient’s interaction with the device and motivating them to complete the task. The graphic applications are processed in the remote desktop [33].

In GTG rehabilitation program, the duration of the single session (45 min) was divided into gait and trunk balance training. The trunk balance training consists of static and dynamic exercises during sitting and standing position (with assistance by the therapist if necessary, according to the ability of the patient to maintain a safe standing position).

Each patient at every session performed at least two exercises between the following:

1. Exercises in static mode with the blocked “seat platform” or “floor platform” where the patient is positioned as still as possible in the Closed Eyes (CE) and Open Eyes (OE) condition;
2. Exercises in dynamic mode with the unblocked “seat platform” or “floor platform” where the patient is positioned as still as possible;
3. Exercises in dynamic mode (the “seat platform” or “floor platform are unblocked along one or more axis).

The difficulty of the exercise was gradually increased over the course of the treatment or even within the same session when the patient’s condition allows it.

The rehabilitation program of both groups was combined with daily conventional physiotherapy including functional task practice, muscle strengthening, speech therapy, conventional gait and balance training, and occupational therapy.

### 2.4. Clinical Evaluation and Instrumental Assessments

Patients were assessed both clinically and instrumentally (balance evaluation) at baseline (T0) and the end (T1) of the rehabilitation program.

As the primary outcome we measured the changes from the baseline of the Berg Balance Scale (BBS) score.

The secondary outcomes measured in the study were gait ability, motor function, performance, activities of daily living, participation, pain, and instrumental balance evaluation. The outcomes that have been chosen, according to the domain of International Classification of Functioning, Disability, and Health (ICF) [35,36], assess body function (Motricity Index (MI) for lower limb, Modified Ashworth Scale (MAS) for lower limb, ID Pain, Numerical Rating Scale (NRS)), activities (modified Barthel Index (mBI), Ambulation Index (AI), Functional Ambulation Classification (FAC), 10-Meter Walk Test (10 MWT), 6-Minute Walk Test (6 MWT), Timed Up and Go Test (TUG), Trunk Control Test (TCT), Berg Balance Scale (BBS) and Tinetti Balance Scale (TIN-B)), and participation (Walking Handicap Scale (WHS)).

The instrumental balance evaluation was performed with the robotic platform Hunova (Movendo). The patients were placed in sitting positions during static and dynamic measurements, and all stabilometric parameters were collected in OE and CE conditions. Starting from the Center of Pressure (CoP) trajectories, these variables related to balance performance were computed: CoP Sway Area (95% confidence ellipse of the statokinesigram, [cm^2^]), CoP Sway path (length of the oscillation path, [cm]), Romberg Index, Antero-Posterior (AP) and Medio-Lateral (ML) CoP oscillation ranges [cm], Ellipse axes ratio [%], and AP and ML CoP Mean velocity [cm/s]. 

Finally, using an accelerometer positioned at the sternal level of the trunk, trunk displacement [deg^2^] and trunk oscillations in AP and ML directions [deg] were calculated.

### 2.5. Safety and Possible Side-Effects during Study Participation

The study did not involve diagnostic or therapeutic procedures of an invasive nature. Therefore, participation did not pose any particular and significant additional risks to those that might be related to performing the ordinary treatment program.

The scales and questionnaires used for clinical assessment are tools widely used in clinical practice and therefore do not present any special risks. The execution of the rehabilitation treatments and assessments always took place in the constant presence of appropriate professional staff to provide the patients with instructions on the activity to be performed and assistance if needed. The G-EO system and the Hunova platform used in the study are low-risk devices and have appropriate certification of compliance with current safety regulations.

### 2.6. Statistical Analysis

The statistical analysis was performed using the SPSS 21 package (IBM, Armonk, NY, USA). The within-group analysis was based on the application of the Wilcoxon Signed Rank test for each clinical and balance outcome registered at T0 and T1.

The between-group differences were analyzed by comparing the percentage increase of each outcome, defined as:

$$\Delta S=\frac{S\left(T1\right)-S\left(T0\right)}{S\left(T0\right)}\;$$

where S is one of the clinical or balance outcomes employed in the study (except for MAS, FAC, AI, NRS and ID PAIN), and S(T0) and S(T1) are the S scores at T0 and T1, respectively. 

The between-group analysis of MAS, FAC, AI, NRS, and ID PAIN scales was conducted by considering the differences between the scores, S(T1)–S(T0), because the minimal value of these scales is 0 or also −1 for ID PAIN and normalization was not thus possible. The Mann–Whitney U test was applied to compare the percentage increase calculated for each group. Statistical significance for each test was set at 0.05.

## 3. Results

### 3.1. Sample

**Figure 2:**
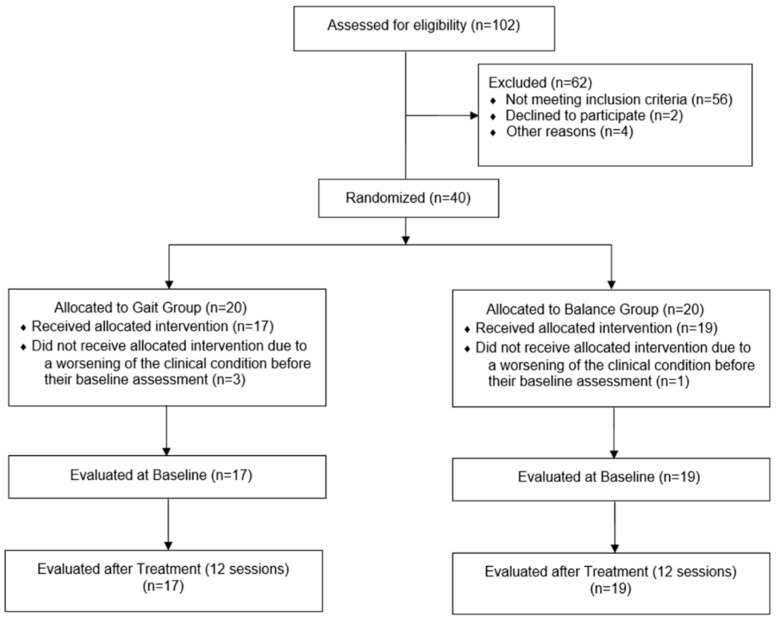
CONSORT Flow Chart.

Between November 2019 and March 2020, 102 subjects were assessed for eligibility (Figure 2). 

The most common reason for exclusion was the age (18 subjects, 29%), cognitive and language deficits (18 subjects, 29%), and a time since stroke outside inclusion criteria range (20 subjects, 32.3%). Less frequently subjects were excluded because of recurrent stroke (2 subjects, 3.2%), or visual deficits (2 subjects, 3.2%). Finally, subjects were excluded because of declining to participate to the study (2 subjects, 3.2%). 

It is noteworthy that, in 35.3% of subjects, more than one exclusion criteria were present.

According to the inclusion criteria, we recruited 40 subacute stroke patients, and they were randomized into GG or GTG, however four patients never received the allocated intervention, due to a worsening of the clinical condition before their baseline assessment. Finally, we considered in the study 36 patients, comprising 21 males and 15 females, aged between 42 and 80 years (mean age 66.36 ± 9.04 years), 22 ischemic and 14 hemorrhagic strokes; 20 with left hemiparesis and 16 with right hemiparesis. Time post the acute event ranged from 25 to 180 days (mean days 126.5 ± 37.9). Table 1 shows the demographic and clinical characteristics at baseline of the GG and GTG. Clinical outcomes at baseline did not show significant differences between the GG and the GTG, so the two groups were comparable at baseline. 

### 3.2. Clinical Outcomes

After treatment, the within-group analysis revealed statistically significant changes in some clinical scales for both the GG and the GTG (Table 2). More specifically, in the GG, we observed a significant statistical improvement in walking (measured by FAC and AI), balance (measured by TIN-B, BBS and TCT), participation (measured by WHS), mobility and self-care (measured by BI), and pain (measured by NRS).

In the GTG, we observed a significant statistical improvement of almost all clinical outcomes: the lower limb muscle strength (measured by MI-AD, MI-KE, MI-HF, the MI-), lower limb muscle tone (measured by MAS-K and MAS-LL), walking (measured by FAC, AI but also 6 MWT, and TUG) balance (measured by TIN-B, BBS and TCT), and mobility and self-care (measured by BI).

Regarding the change from baseline and, therefore, the between-group analysis, we no found statistically significant differences between GG and GTG in primary and secondary clinical outcomes (Table 2), so there is not a group that improves more than another one.

### 3.3. Instrumental Outcomes

Balance variables at baseline did not show significant differences between the GG and the GTG both in static and dynamic conditions, so the two groups were comparable at baseline.

Regarding the balance evaluation in static condition after rehabilitation, no difference was detected in the within-group analysis. In the between-group analysis we found a significant difference only in CoP Mean velocity ML OE (*p* = 0.047) because GTG improves more than GG. These results are shown in Table 3.

Regarding the balance evaluation in the dynamic condition, after rehabilitation the within-group analysis revealed statistically significant changes only in GTG (Table 4). In particular, we observed an improvement in CoP Sway Area in closed eyes (*p* = 0.017), in AP CoP oscillations in closed eyes condition (*p* = 0.023), in ML CoP oscillations in open eyes condition (*p* = 0.012), in ML CoP oscillations in closed eyes condition (*p* = 0.041), in AP CoP mean velocity in closed eyes condition (*p* = 0.036), in trunk displacement in open and closed eyes condition (*p* = 0.009 and *p* = 0.004, respectively), in AP trunk oscillations in open and in closed eyes condition (*p* = 0.006 and *p* = 0.022, respectively), and ML trunk oscillations in open eyes (*p* = 0.026).

**Figure 3:**
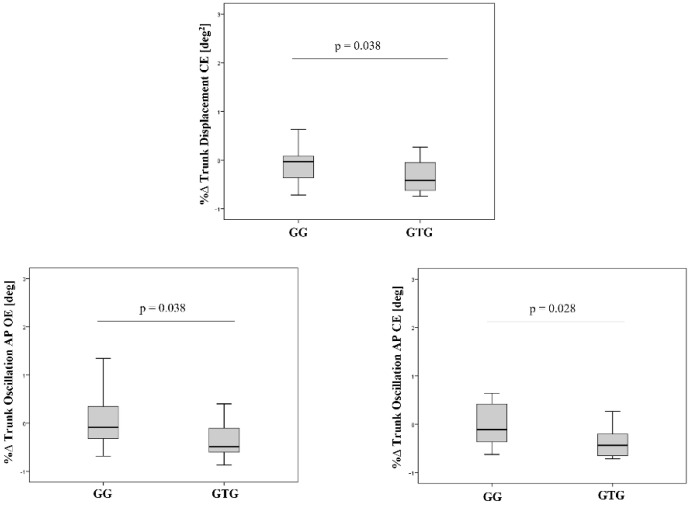
Dynamic condition results: between-group statistical analysis (comparison of the percentage changes—%∆GaitGroup vs. %∆Gait/TrunkGroup—obtained in the two groups).

Furthermore, Figure 3 shows the statistically significant improvement between the two groups for the percentage changes of the trunk displacement and AP trunk oscillation in both open and closed eyes conditions.

## 4. Discussion

In this randomized controlled trial, we evaluated the efficacy of a combined robotic walking and trunk rehabilitation treatment compared to robotic gait training alone in a group of subacute stroke patients.

Patients who receive a combination of robotic gait and trunk balance training achieved the same benefits as patients who receive robotic gait training alone. The primary outcome and almost all other clinical and instrumental measurements revealed similar effects in terms of improved balance ability after treatment in both groups. Therefore, we can deduce that patients of both groups improved equally. Furthermore, robotic treatment, as demonstrated in our previous studies conducted in a group of subacute stroke patients [37], led to a significant improvement in walking endurance. The idea is that the robots offer intensive, highly repetitive and symmetrical training that provides significant improvements in ambulation [22,23,37,38].

Nonetheless, some interesting differences between the two groups were observed in the present study: lower limb muscle strength and tone were significantly improved only in the GTG, hence the mechanical perturbation of the robotic platform during trunk balance training probably causes patients to improve lower limb muscle activity and strength [33].

Accordingly, we hypothesize that improvements in muscle strength and tone may also explain the significant increase in gait endurance that occurs only in the GTG.

Furthermore, our results are in line with those of other studies, in which the unstable surface of the robotic platform induced an improvement in gait endurance [33,39].

Another important result concerns the trunk control; the combined gait/trunk robotic treatment in the GTG provided a significant reduction in trunk oscillations and trunk displacement, both in open-eyes and closed-eyes conditions, during dynamic exercises, more than in the GG.

Combined gait/trunk training pilots patients to have an overall reduction in trunk instability and leads them to have better control during external perturbations. In conclusion, patients acquire the ability to stabilize the spine and the trunk muscles. These data are consistent with other findings recently published by other researchers, in which stroke patients showed a significant improvement in medio-lateral trunk behavior when treated with the robotic platform [33].

Stroke patients have balance and walking problems, mainly due to asymmetric motor impairment and posture [20,40]. In particular, sitting balance is a fundamental prerequisite for achieving standing balance and thus independent daily living, and improving patients’ quality of life [41,42,43,44]; therefore, trunk balance rehabilitation is an important component of rehabilitation treatment [45].

Usually, trunk balance rehabilitation can be achieved by conventional balance training or gait training [2]. To date, innovative robotic devices are available for balance treatment and dynamic balance recovery; the latter is very important for achieving safer gait. Previous studies have evaluated the improvement in balance after treatment using only clinical scales [46,47]. Furthermore, to our knowledge, only a few and incomplete studies have considered the effect of robot gait training on balance using instrumental measures [31]. Our study is the first randomized clinical trial combining robotic gait and trunk rehabilitation treatment and comparing it with robotic gait training alone in a group of stroke patients that aims to find rehabilitation training methods that maximize the benefits of the robotic-assisted devices. Our results suggest that the addition of a robotic platform to robotic walking treatment provides more intensive and controlled trunk training that positively affects muscle tone and lower limb strength. Although only slight improvements were obtained, from the patients’ perspective it is important to take advantage of every opportunity to improve function, and these preliminary results suggest the use of these devices in clinical practice and the need to treat and follow patients for a longer period as well.

## 5. Conclusions

In conclusion, this randomized controlled trial demonstrates that the addition of a robotic platform to robotic gait treatment provides more intensive and controlled trunk training that positively influences lower limbs muscle tone and strength and trunk stability. However, the present work has limitations, including the lack of follow-up, the sample size, which should be increased, and the severe impairment of the patients.

In addition, instrumental assessment of the trunk was only possible in sitting position precisely because of the severity of the patients; future research on patients with mild or moderate impairment could help clinicians and researchers learn more about the effects of combined robotic treatment of gait and trunk.

Furthermore, subacute stroke patients undergoing an inpatient rehabilitation program, have more severe impairment than patients undergoing a home or outpatient rehabilitation program; therefore, if we have information on long-term treatment or long follow-up of the patients, we can evaluate the effects of integrated robotic gait/trunk treatment even in the standing condition.

## Figures and Tables

**Table 1:** Characteristics of the Sample and Clinical Outcomes at T0 (N = 36).

**A:** Characteristics			
	GG	GTG	*p*-value
	*n* (%) Mean ± SD		
Subjects	17 (47.20)	19 (52.80)	
Gender. Male/Female	9 (52.94)/8 (47.06)	12 (63.16)/7 (36.84)	0.616
Age (years)	66.64 ± 9.61	66.11 ± 8.76	0.778
Time post the acute event (days)	119.9 ± 38.9	134.3 ± 36.1	0.129
Aetiology. Ischemic/Haemorrhagic	9 (52.94)/8 (47.06)	13 (68.42)/6 (31.58)	0.265
Lesion Side. Left/Right	10 (58.82)/7 (41.18)	10 (52.64)/9 (47.36)	0.754
**B:** Clinical Outcomes at T0			
	GG	GTG	*p*-value
	Median [25th;75th percentiles]		
MI-LL	42 [27–66]	53 [42–75]	0.285
MAS-LL	2 [1–4]	2 [0–4]	0.531
FAC	0 [0–1]	1 [0–2]	0.165
TIN-B	8 [6–9]	11 [4–14]	0.219
BBS	13 [8–27]	22 [8–38]	0.452
TCT	61 [37–62]	61 [37–74]	0.639
WHS	1 [1–1]	1 [1–2]	0.066
10 MWT velocity m/s	0.44 [0.33–0.67]	0.30 [0.17–0.42]	1.000
6 MWT distance (m)	117 [44–154]	72 [25–144]	0.539
AI	1 [0–1]	1 [1–2]	0.076
TUG time (s)	24 [16–36]	29 [22–47]	0.373
BI	36 [22–50]	42 [26–67]	0.330
NRS	4 [1–6]	3 [0–5]	0.415
ID PAIN	1 [0–2]	0 [−1–2]	0.232

Abbreviations: GG—Gait Group; GTG—Gait/Trunk Group; T0—before the treatment; T1—at the end of the treatment; MI-LL—Motricity Index affected Lower Limb; MAS-LL—Modified Ashworth Scale affected Lower Limb; FAC—Functional Ambulatory Classification; TIN-B—Tinetti Scale Balance; BBS—Berg Balance Scale; TCT—Trunk Control Test; WHS—Walking Handicap Scale; 10 MWT—Ten-Meter Walking Test; 6 MWT—Six-Minute Walking Test; AI—Ambulation Index; TUG—Timed Up and Go Test; BI—Barthel Index; NRS—Numerical Rating Scale.

**Table 2:** In the table are reported the clinical scale values (medians and interquartile ranges) at T0 and T1, and its within-group statistical analysis results (T0 vs. T1 evaluation) for both the GG and GTG group; and the *p* values referring to the comparisons of the percentage changes between-group statistical analysis results. In bold are the statistically significant values.

	GG			GTG			*p* Value (%∆_GG vs. GTG)
	T0 Median (IQR)	T1 Median (IQR)	*p* Value (T0 vs. T1)	T0 Median (IQR)	T1 Median (IQR)	*p* Value (T0 vs. T1)	
**MI-AD**	14 (9–19)	14 (9–25)	0.068	14 (14–25)	25 (14–33)	**0.010**	0.346
**MI-KE**	14 (9–25)	19 (12–25)	0.059	14 (14–25)	25 (14–33)	**0.005**	0.100
**MI-HF**	14 (9–25)	25 (9–25)	0.126	19 (14–25)	25 (19–33)	**0.011**	0.433
**MI-LL**	42 (27–66)	58 (32–70)	0.065	53 (42–75)	72 (47–91)	**0.002**	0.156
**MAS-H**	0 (0–2)	0 (0–1)	0.083	0 (0–1)	0 (0–0)	0.096	0.754
**MAS-K**	0 (0–1)	0 (0–1)	0.582	0 (0–1)	0 (0–0)	**0.038**	0.490
**MAS-A**	1 (0–2)	1 (1–2)	0.558	1 (0–2)	1 (0–2)	0.102	0.802
**MAS-LL**	2 (1–4)	2(1–3)	0.277	2 (0–4)	1 (0–3)	**0.026**	0.552
**FAC**	0 (0–1)	1 (1–3)	**0.004**	1 (0–2)	2 (1–4)	**0.000**	0.684
**TIN-B**	8 (6–9)	11 (7–14)	**0.021**	11 (4–14)	12 (8–16)	**0.004**	0.975
**BBS**	13 (8–27)	24 (10–36)	**0.007**	22 (8–38)	31 (12–48)	**0.003**	0.900
**TCT**	61 (37–62)	62 (37–80)	**0.018**	61 (37–74)	74 (49–100)	**0.009**	0.778
**WHS**	1 (1–1)	1 (1–2)	**0.010**	1 (1–2)	2 (1–4)	**0.020**	0.616
**10 MWT**	0.44 (0.33–0.67)	0.49 (0.30–0.75)	0.500	0.30 (0.17–0.42)	0.37 (0.26–0.53)	0.575	0.093
**6 MWT**	117 (44–154)	175 (51–328)	0.068	72 (25–144)	119 (71–195)	**0.007**	0.839
**AI**	1 (0–1)	3 (1–5)	**0.001**	1 (1–2)	3 (3–3)	**0.000**	0.616
**TUG**	24 (16–36)	17 (7–35)	0.593	29 (22–47)	21 (14–39)	**0.015**	1.000
**BI**	36 (22–50)	52 (44–71)	**0.001**	42 (26–67)	68 (45–80)	**0.000**	0.684
**NRS**	4 (1–6)	2 (0–4)	**0.018**	3 (0–5)	2 (0–4)	0.178	0.300
**ID PAIN**	1 (0–2)	1 (−0–1)	0.174	0 (−1–2)	0 (0–1)	0.796	0.397

Abbreviations: GG—Gait Group; GTG—Gait/Trunk Group; T0—before the treatment; T1—at the end of the treatment; MI-AD—Motricity Index affected Ankle Dorsiflexion; MI-KE—Motricity Index affected Knee Extension; MI-HF—Motricity Index affected Hip Flexion; MI-LL—Motricity Index affected Lower Limb; MAS-H—Modified Ashworth Scale affected Hip; MAS-K—Modified Ashworth Scale affected Knee; MAS-A—Modified Ashworth Scale affected Ankle; MAS-LL—Modified Ashworth Scale affected Lower Limb; FAC—Functional Ambulatory Classification; TIN-B—Tinetti Scale Balance; BBS—Berg Balance Scale; TCT—Trunk Control Test; WHS—Walking Handicap Scale; 10 MWT—Ten-Meter Walking Test; 6 MWT—Six-Minute Walking Test; AI—Ambulation Index; TUG—Timed Up and Go Test; BI—Barthel Index; NRS—Numerical Rating Scale.

**Table 3:** In the table are reported balance outcomes in the sitting position and static condition (medians and interquartile ranges) at T0 and T1, and its within-group statistical analysis results (T0 vs. T1 evaluation) for both the GG and GTG group; and the *p* values referring to the comparisons of the percentage changes between-group statistical analysis results. In bold the statistically significant values.

	GG			GTG			*p* Value (%∆_GG vs. GTG)
	T0 Median (IQR)	T1 Median (IQR)	*p* Value (T0 vs. T1)	T0 Median (IQR)	T1 Median (IQR)	*p* Value (T0 vs. T1)	
**CoP sway Area OE [cm^2^]**	0.2 (0.1–1.2)	0.2 (0.1–0.5)	0.756	0.2 (0.2–0.5)	0.2(0.1–1.0)	0.959	0.897
**CoP sway Area CE [cm^2^]**	0.2 (0.0–0.6)	0.1 (0.05–0.3)	0.605	0.1(0–1.2)	0.1(0.0–0.4)	0.469	0.669
**CoP sway Path OE [cm]**	8.4 (3.5–13.5)	8.2 (4.4–14.65)	0.438	9.5 (5.5–12.3)	7.9(6.6–11.9)	0.796	0.287
**CoP sway Path CE [cm]**	7.3 (3.4–10.2)	7.6 (4.3–11.2)	0.959	7.7 (4.0–11.3)	7.2 (6.0–8.8)	0.569	1.000
**Romberg Index**	2.7 (1.6–5.2)	1.7 (1.1–4.5)	0.469	2.5 (1.5–4.0)	2.6 (1.2–13.3)	0.535	0.491
**CoP Oscillation AP OE [cm]**	0.7 (0.3–1.3)	0.7 (0.4–1.1)	0.501	0.9 (0.7–1.4)	0.6 (0.3–1.1)	0.408	0.184
**CoP Oscillation AP CE [cm]**	0.5 (0.3–0.8)	0.6 (0.4–1.2)	0.234	0.9 (0.5–1.9)	0.7 (0.4–1.7)	0.352	0.184
**CoP Oscillation ML OE [cm]**	0.7 (0.3–1.2)	0.6 (0.4–0.9)	0.959	0.8 (0.5–1.4)	0.5 (0.4–1.0)	0.352	0.515
**CoP Oscillation ML CE [cm]**	0.7 (0.3–1.1)	0.6 (0.3–0.9)	0.379	0.5 (0.4–1.4)	0.6 (0.4–0.9)	0.959	0.724
**Ellipse axes ratio OE [%]**	45.5 (41.4–61)	50.7 (34.6–57.3)	0.756	50.1 (32.2–63.6)	46.0 (40.1–50.4)	0.569	0.809
**Ellipse axes ratio CE [%]**	44.0 (36.9–57.6)	40.7 (28.4–55.8)	0.234	52.5 (46.6–66.3)	48.5 (40.4–53.8)	0.109	0.616
**CoP Mean velocity AP OE [cm/s]**	0. 3 (0.1–0.5)	0.3 (0.2–0.5)	0.756	0.4 (0.2–0.5)	0.3 (0.2–0.4)	0.717	0.361
**CoP Mean velocity AP CE [cm/s]**	0.3 (0.1–0.4)	0.3 (0.1–0.4)	0.836	0.3 (0.2–0.5)	0.3 (0.2–0.3)	0.650	0.402
**CoP Mean velocity ML OE [cm/s]**	0.29 (0.17–0.54)	0.32 (0.21–0.56)	0.379	0.35(0.25–0.47)	0.32 (0.26–0.54)	0.600	**0.047**
**CoP Mean velocity ML CE [cm/s]**	0.3 (0.2–0.4)	0.3 (0.2–0.4)	0.918	0.3 (0.2–0.4)	0.3 (0.2–0.4)	0.753	0.491
**Trunk Displacement OE [deg^2^]**	0.04 (0.03–0.1)	0.04 (0.03–0.05)	0.877	0.04 (0.03–0.05)	0.04 (0.03–0.06)	0.733	0.520
**Trunk Displacement CE [deg^2^]**	0.03 (0.03–0.04)	0.03 (0.03–0.04)	0.605	0.03 (0.03–0.05)	0.03 (0.03–0.04)	0.233	0.572
**Trunk Oscillation AP OE [deg]**	3.0 (1.6–4.0)	3.3 (2.3–4.3)	0.918	3.4 (2.2–7.4)	3.3 (1.8–6.5)	0.691	0.545
**Trunk Oscillation AP CE [deg]**	2.9 (1.3–3.5)	3.3 (2.2–5.3)	0.056	2.9 (1.5–5.7)	3.4 (2.5–4.4)	0.650	0.264
**Trunk Oscillation ML OE [deg]**	1.8 (0. 9–4.1)	2.2 (1.2–4.4)	0.352	1.8 (1.3–2.3)	2.2 (1.2–3.9)	0.955	0.520
**Trunk Oscillation ML CE [deg]**	1.6 (0.9–4.6)	1.9 (1.2–2.8)	0.959	2.2 (1.0–3.1)	1.9 (1.4–2.9)	0.910	1.000

Abbreviations: GG—Gait Group; GTG—Gait/Trunk Group; T0—before the treatment; T1—at the end of the treatment; IQR—interquartile ranges; CoP—Center of Pressure; OE—Open Eyes; CE—closed eyes; AP—Antero-Posterior; ML—Medio-Lateral.

**Table 4:** In the table are reported balance outcomes in Sitting Position and Dynamic Condition (medians and interquartile ranges) at T0 and T1, and its within-group analysis results (T0 vs. T1 evaluation) for both GG and GTG group; and the *p* values referring to the comparisons of the percentage changes between-group statistical analysis results. In bold the statistically significant values.

	GG			GTG			
	T0 Median (IQR)	T1 Median (IQR)	*p* Value (T0 vs. T1)	T0 Median (IQR)	T1 Median (IQR)	*p* Value (T0 vs. T1)	*p* Value (%∆_GG vs. GTG)
**CoP sway** **Area OE [cm^2^]**	125.3 (18.5–573.4)	235.7 (57.6–383.5)	0.717	392.4 (43.2–512.1)	79.7 (5.6–426.7)	0.281	0.140
**CoP sway** **Area CE [cm^2^]**	170 (43.7–431.7)	137.5 (50.2–327.1)	0.918	301.3 (69.3–677.8)	92 (4.3–248.1)	**0.017**	0.086
**CoP sway Path OE [cm]**	41.3 (21.1–82.4)	50 (35.8–75.2)	0.501	67.7 (22.1–85.0)	43 (10.2–72.5)	0.156	0.140
**CoP sway Path CE [cm]**	50.8 (28.8–82.2)	37.4 (29–74.6)	0.836	68 (42.9–99.6)	31.5 (9.1–72.3)	0.078	0.188
**Romberg Index**	1.4 (0.6–3.6)	3.5 (1.7–5.1)	0.098	2.3 (1.0–5.0)	1.8 (0.6–4.7)	0.650	0.247
**CoP Oscillation AP OE [** **cm** **]**	10.7 (5–15.1)	11.1 (7.4–17.5)	0.569	14.4 (6.8–20.3)	7.1 (2.5–18.3)	0.112	0.101
**CoP Oscillation AP CE [** **cm** **]**	9.5 (5.2–18.2)	11.9 (6.8–15.1)	1.000	14.1 (7.1–17.6)	7.7 (3.2–10.6)	**0.023**	0.078
**CoP Oscillation ML OE [** **cm** **]**	14.3 (6.2–19.8)	18.4 (10.3–21.2)	0.501	19.7 (9.4–29.3)	14.1 (2.5–18.3)	**0.012**	0.072
**CoP Oscillation ML CE [** **cm** **]**	18.9 (12.3–24.4)	17 (7.8–24)	0.756	21.8 (9.9–28.2)	13.5 (3.7–18)	**0.041**	0.216
**Ellipse axes ratio OE [%]**	33.3 (14.3–44.8)	32.9 (17.7–37.5)	0.605	24.9 (18.6–39.3)	29.7 (23.2–40.9)	0.427	0.545
**Ellipse axes ratio CE [%]**	33.7 (20.1–46.2)	28.4 (19.3–36.7)	0.352	37.4 (24.9–50.5)	39.1 (26.3–50.3)	0.427	0.830
**CoP Mean velocity AP OE [cm/s]**	1.5 (0.7–2.2)	1.9 (0.7–2.2)	0.570	1.5 (0.7–2.8)	1.2 (0.3–2.3)	0.140	0.299
**CoP Mean velocity AP CE [cm/s]**	1.2 (0.8–2.2)	1.4 (0.8–2.4)	0.865	1.7 (0.8–2.6)	0.9 (0.3–1.5)	**0.036**	0.140
**CoP Mean velocity ML OE [cm/s]**	2.1 (0.6–3.4)	2.2 (1.2–4)	0.776	2.9 (0.7–4.9)	2.2 (0.3–3.3)	0.233	0.830
**CoP Mean velocity ML CE [cm/s]**	2.4 (1.2–3.3)	1.8 (0.8–3.2)	0.820	3.1 (2.1–4.0)	1.4 (0.2–3.9)	0.125	0.599
**Trunk Displacement OE [deg^2^]**	0.12 (0.08–0.20)	0.12 (0.09–0.14)	0.379	0.123 (0.084–0.204)	0.120 (0.090–0.141)	**0.009**	0.131
**Trunk Displacement CE [deg^2^]**	0.09 (0.06–0.14)	0.09 (0.06–0.13)	0.326	0.092 (0.062–0.140)	0.091 (0.060–0.138)	**0.004**	**0.038**
**Trunk Oscillation AP OE [deg]**	12.6 (7–19.2)	12.8 (8.6–15.3)	0.535	14.6 (6.5–18.5)	7.1 (3.2–11.6)	**0.006**	**0.038**
**Trunk Oscillation AP CE [deg]**	11.8 (7–16.4)	11 (5.6–16.2)	0.642	12.4 (7.6–16.5)	6 (3.4–10.5)	**0.022**	**0.028**
**Trunk Oscillation ML OE [deg]**	11.5 (6.9–20.8)	11.3 (7.5–17)	0.877	12 (9.2–18.3)	6 (2.3–10.1)	**0.026**	0.101
**Trunk Oscillation ML CE [deg]**	9.6 (6–15.4)	9.8 (3.9–17.7)	0.642	10.2(6.1–21.9)	4.9 (2–11.4)	0.056	0.110

Abbreviations: GG—Gait Group; GTG—Gait/Trunk Group; T0—before the treatment; T1—at the end of the treatment; IQR—interquartile ranges; CoP—Center of Pressure; OE—Open Eyes; CE—closed eyes; AP—Antero-Posterior; ML—Medio-Lateral.

## Data Availability

Data are stored in a password-protected PC located in the Movement Laboratory of Fondazione Don Carlo Gnocchi.
